# Use of *Leishmania major* parasites expressing a recombinant *Trypanosoma cruzi* antigen as live vaccines against Chagas disease

**DOI:** 10.3389/fmicb.2022.1059115

**Published:** 2022-11-29

**Authors:** Catherine W. Cai, Anne O’Shea, Christopher S. Eickhoff, Hongjie Guo, Warren G. Lewis, Stephen M. Beverley, Daniel F. Hoft

**Affiliations:** ^1^Department of Molecular Microbiology & Immunology, Saint Louis University School of Medicine, Saint Louis, MO, United States; ^2^Division of Infectious Diseases, Allergy and Immunology, Department of Internal Medicine, Saint Louis University School of Medicine, Saint Louis, MO, United States; ^3^Department of Molecular Microbiology, Washington University School of Medicine in St. Louis, Saint Louis, MO, United States

**Keywords:** Chagas, leishmaniasis, vaccine, parasite, live vector

## Abstract

**Introduction:**

*Trypanosoma cruzi* is the protozoan parasite causing Chagas disease, a Neglected Tropical Disease that affects 8 million people and causes 12,000 deaths per year, primarily because of cardiac pathology. Effective vaccination for *T. cruzi* remains an elusive goal. The use of a live vaccine vector, especially one that mimics the pathogen target, may be superior to the use of recombinant protein or DNA vaccine formulations.

**Methods:**

We generated recombinant *Leishmania major*, a related trypanosomatid parasite, as a vaccine vehicle to express the immunogenic *T. cruzi trans*-sialidase (TS) antigen. The induction of T cell and antibody responses, as well as *T. cruzi* protective immunity generated by these vaccines were assessed in vivo.

**Results:**

We demonstrate that mice inoculated with these recombinant TS-expressing *L. major* parasites mount T cell and antibody responses directed against TS and are protected against future *T. cruzi* infection. We also show that the partially attenuated *dhfr-ts- CC1 L*. major strain, previously found to induce protective immunity to virulent *L. major* infection without causing pathology, can also be engineered to express the TS antigen. This latter recombinant may represent a safe and effective option to explore for ultimate use in humans.

**Discussion:**

Altogether, these data indicate that *L. major* can stably express a *T. cruzi* antigen and induce *T. cruzi*-specific protective immunity, warranting further investigation of attenuated Leishmania parasites as vaccine.

## Introduction

Chagas disease results from chronic infection with the protozoan parasite *Trypanosoma cruzi*. The disease is classified as a Neglected Tropical Disease by the World Health Organization for its impact on global health and disproportionate burden on developing economies. Eight million people are infected, primarily in Latin America where it is endemic, and another 100 million are at risk (Bern and Montgomery, 2009). Approximately 30% of chronically infected people develop life-threatening cardiac or gastrointestinal pathologies, accounting for more than 12,000 deaths every year (Montgomery et al., 2014). Available drug therapies including benznidazole and nifurtimox have poor efficacy in the chronic phase and are complicated by a number of undesirable side effects that reduce adherence (Bern, 2011). In addition, costs of these medications can be prohibitive, particularly for impoverished communities most at risk (Manne et al., 2013). Because of the significant effects on human health, lack of optimal therapies, and the associated financial impacts, vaccines are an economical approach to addressing this problem (Lee et al., 2010).

We and others have previously investigated the *T. cruzi trans*-sialidase (TS) protein as a potential antigen for Chagas vaccines (Costa et al., 1998; Hoft et al., 2007; Fontanella et al., 2008; Eickhoff et al., 2011; Bontempi et al., 2015). We have shown that vaccination with TS DNA or recombinant TS adjuvanted with CpG can protect mice from mortality following a virulent challenge (Hoft et al., 2007; Eickhoff et al., 2011). The TS antigen is encoded by a large family of hundreds of related genes in the *T. cruzi* genome (Frasch, 2000), only some of which exhibit catalytic neuraminidase activity that transfers sialic acid residues from the host cell surface to the parasite surface, thus assisting virulence and evasion of host immune responses (Alves and Colli, 2007). Inhibition of TS enzymatic activity reduces *T. cruzi* pathogenicity, indicating that trans-sialylation has a critical virulence function (Freire-de-Lima et al., 2010). Importantly, the consensus domain of this enzyme is highly conserved, making it an attractive target for vaccine development (Buschiazzo et al., 2002).

Despite several partially efficacious experimental vaccines studied in mice, none have conferred immunity as robust as the protection that naturally arises from persistent, low-level infection, known as concomitant immunity. Adoptive transfer of CD8+ T cells from mice repeatedly subjected to low-level *T. cruzi* infections are sufficient to protect highly susceptible naïve mice from parasite challenges (Sullivan et al., 2015) emphasizing the strength of concomitant immunity. Similarly, inoculation with live vaccines often affords stronger protection than other formulations.

A naturally attenuated strain of *T. cruzi* has been tested as a Chagas disease vaccine in animal models (Basombrio et al., 1993; Perez Brandan et al., 2011), but the possibility of reversion to virulence *in vivo* limits the development of such strains for human vaccination. In *Leishmania major,* a related trypanosomatid parasite that normally causes an endemic skin and organ disease known as leishmaniasis, several gene knockout parasites have been generated which have lost the ability to cause pathology, but persist and induce protective immunity to varying extents. One of the first studied was attenuated through targeted deletion of the dihydrofolate reductase-thymidylate synthase gene (*DHFR-TS*) (Titus et al., 1995). This homozygous knock-out strain is auxotrophic for thymidine, and is therefore unable to replicate *in vivo,* where it is progressively lost over a period of 60 days. Normally, thymidine levels are tightly controlled *in vivo*, at levels insufficient to rescue *dhfr-ts^−^* growth (Titus et al., 1995). However, by providing near-toxic levels of thymidine by continuous infusion, rescue of *dhfr-ts^−^* growth could be obtained (Titus et al., 1995). Though these parasites do not cause pathology, they induce strong *L. major* immunity protective against future virulent challenges. The safety of this avirulent *dhfr-ts^−^ L. major* strain has been well-characterized (Titus et al., 1995). While *dhfr-ts^−^* strains were originally constructed using antibiotic resistance markers including the *NEO* marker which mediates resistance to the antileishmanial paromomycin (Gueiros-Filho and Beverley, 1994), marker-free parasites can be obtained by a counter-selection approach (Gueiros-Filho and Beverley, 1996), and more recently, CRISPR/Cas9 mutagenesis has been adapted to generate marker free parasites at other loci (Zhang et al., 2020).

Attempts to generate *dhfr-ts^−^ T. cruzi* have been challenged by the inability to generate homozygous deletions (Perez Brandan et al., 2011). Because *L. major* infects phagocytic cells including macrophages and dendritic cells, which function as important antigen-presenting cells (APCs) (Ng et al., 2008), avirulent *L. major* parasites could serve as live vectors for the delivery of recombinant vaccine antigens (Breton et al., 2005). In addition, this strain induces a primarily Th1 response, which is known to be protective against *T. cruzi* infection (Brodskyn et al., 2000; Hoft et al., 2000). Thus, we asked whether we could use the pre-existing *dhfr-ts^−^* CC1 *L. major* as a live vehicle for the delivery of Chagas vaccine antigens by genetically engineering the parasites to express an immunogenic *T. cruzi* protein.

Here we tested vaccinations against *T. cruzi* infection using live *Leishmania* engineered to express an active *T. cruzi* TS gene following integration into the rRNA locus using the pIR1SAT platform (Capul et al., 2007a,b). *T. cruzi* TS is encoded by a large gene family, only some of which bear catalytic activity (El-Sayed et al., 2005); the TS used here was chosen as it is a highly immunogenic virulence factor containing both immunodominant CD4 and CD8 epitopes (Hoft et al., 2007; Cai et al., 2016; Eickhoff et al., 2016). We created recombinant wild type and attenuated *L. major* (Lm) expressing various forms of the TS catalytic domain with or without the secretory domains, previously shown to induce similar protective immunity as the full protein (Fujimura et al., 2001). We then infected mice with these rTS-*Lm* vaccine delivery lines and analyzed the ability of these vaccines to induce protective *T. cruzi* immunity by measuring TS-specific T cell and antibody responses, and survival following *T. cruzi* challenge. We demonstrate that recombinant *L. major* parasites expressing the *T. cruzi* TS antigen effectively induce TS-specific T cell and antibody responses known to be protective against *T. cruzi*-related mortality. These data demonstrate for the first time that *Leishmania* parasites can serve as an effective antigen-delivery vehicle for Chagas disease vaccines. Importantly, attenuated *Lm* expressing *T. cruzi* antigens could act as dual vaccines against both *Leishmania major* and *Trypanosoma cruzi*, two globally important infectious diseases with notable geographical overlap. Thus, recombinant live parasite vaccination could represent a practical and efficient approach and merits further investigation and development.

## Materials and methods

### Molecular constructs

**Figure 1:**
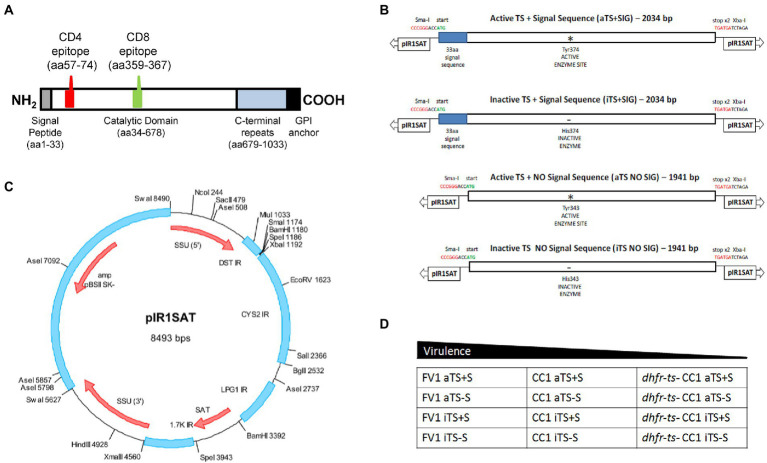
Generation of recombinant TS-expressing *Leishmania* parasites. **(A)** Schematic of *T.cruzi trans*-sialidase (TS154) showing key domains and the location of the known immunodominant I-A^d^-restricted CD4 epitope (p7) and H-2K^d^-restricted CD8 epitope (TSKd1). **(B)** Schematic showing the four TSA constructs tested here: WT or active secreted TS (aTS+S), active nonsecreted TS (aTS−S), inactive secreted TS (iTS+S), inactive nonsecreted TS (iTS−S). Inactive TS versions were generated by point mutation in the enzymatic domain as indicated; non-secreted versions were generated by deleting a 33-amino acid signal sequence at the N-terminus. **(C)** These TS inserts were then cloned into a pIR1SAT vector, optimized for transfection of *L. major*. **(D)** The pIR1SAT shuttle plasmid expressing various forms of TS were used to transfect virulent FV1 and CC1 *L. major* parasites, as well as the avirulent *dhfr-ts^−^* CC1 *L. major* strain.

We assembled by standard PCR and molecular cloning procedures four different constructs expressing TS for this work, introducing the indicated ORFs into the “A” (SmaI/XbaI) site of pIR1SAT, chosen as this site is more highly expressed in amastigotes (Capul et al., 2007a,b) as summarized in Figure 1.; the starting TS gene was TS154/13(Costa et al., 1998). ORFs were amplified with primers bearing an added 5’ SmaI site and a CCACC sequence to enhance expression, and a 3’ XbaI site after the stop codon. WT ORF constructs used the native ATG while the non-secreted constructs removed the first 33 amino acids bearing the predicted signal sequence; these were the aTS+S or aTS−S constructs, respectively. From these, catalytically inactive mutants were generated by site specific mutagenesis, introducing a Y374H mutation into WT (Y343H in the TS−S construct), yielding the iTS+S or iTS−S constructs, respectively. The final DNAs were digested with SmaI and XbaI and the TS-bearing fragments isolated for introduction into SmaI/XbaI-digested pIR1SAT (Figure 1). The relevant regions of all constructs were confirmed by sequencing before use, and the functionality (secretion or activity) was confirmed in transfectants as described in the text. Constructs were digested with SwaI to expose the rRNA segment termini for homologous integration into the parasite prior to use.

### *Leishmania* culture and transfection

*L. major* strain Friedlin V1 (MHOM/IL/80/ Friedlin) was grown at 26°C in M199 medium (U.S. Biologicals) containing 10% heat-inactivated fetal bovine serum and other supplements as described (Kapler et al., 1990). *Leishmania* cells were transfected with SwaI-cut DNA by electroporation using high voltage protocol (Robinson and Beverley, 2003). Following transfection, cells were allowed to grow for 16–24 h in M199 medium and then plated on semisolid media containing 1% Nobel agar (fisher) and 100 μg/ml nourseothricin. Individual colonies were picked and grown in liquid medium in same drug concentration as used in plates. Clones were maintained in selective medium and then removed from selection for one passage prior to experiments.

### Flow cytometry

For flow cytometric detection of TS expression, parasites were pelleted, washed with PBS, and fixed by incubation with 1% paraformaldehyde in PBS for 5 min. The fixed parasites were washed again and incubated on ice in methanol for 30 min to permeabilize the cells. After permeabilization, the cells were washed and incubated with sera from *T. cruzi*-infected animals for 30 min in the dark at 4°C, then washed and incubated with a goat anti-mouse IgG-Alexa Fluor 647 antibody for 30 min in the dark at 4°C. Cells were washed and resuspended in PBS containing 2% fetal bovine serum, and stored protected from light and heat until acquired on a BD FACS Canto II machine. Results were analyzed using FlowJo v7 software (Tree Star, Inc.).

### Western blot

Laemmli buffer was added to parasite lysates and supernatants previously concentrated using 10G spin concentrators (Pierce), and the samples were added to the wells of a 10% acrylamide gel. Recombinant TS protein was used in the assay as a positive control, and Spectra Multicolor Broad Range Protein Ladder was added as a reference (Thermo Fisher). After SDS-PAGE separation and transfer, the nitrocellulose membrane was blocked with 1% milk/PBS for 30 min and then incubated overnight with a rabbit anti-TS antibody diluted in 1% milk/PBS at 4°C. After washing, the blot was incubated with donkey anti-rabbit-HRP diluted in PBS for 1 h, and rinsed. Finally, the membrane was soaked in developer solution with shaking. Once bands appeared, the solution was decanted and the gel rinsed with H_2_O.

### TS enzyme activity assay

To generate parasite lysates and supernatants, parasites were cultured overnight in M199 medium at 1 × 10^6^ parasites per well of a 6-well plate. The following day, the parasites were pelleted by centrifugation. Supernatants were aspirated and concentrated using 10 g spin concentrators (Pierce), and the remaining parasite pellet was lysed. TS enzymatic activity was measured as described previously (Schrader and Schauer, 2006; Eickhoff et al., 2016). Supernatants and lysates were incubated with 1 mM 3′-sialyllactose, a donor substrate simulating the sialic acid residues bound to host cell glycoproteins; 0.5 mM 4-methylumbelliferyl-β-D-galactoside (MUGal), a sialyl acceptor substrate; and 100 mM PIPES medium at 20–26°C for 45 min. Samples were added to spin columns containing previously equilibrated Q-sepharose ion exchange resin and washed and eluted HCl. The flow-through was neutralized with Glycine/NaOH buffer. Functional TS activity creates 4-methylumbelliferyl-β-D-sialylgalactoside, the sialylated form of MUGal from the substrates, which results in a fluorescent substrate following acid hydrolysis. The resulting fluorescence correlates with TS activity and is measured by a microplate reader.

### Mice, parasites, vaccinations, and challenges

All experiments were performed using wild-type BALB/c mice purchased from NCI Charles River Laboratories (Frederick, MD). All mice were housed under specific pathogen free conditions under the care of licensed veterinarians and animal technicians from the Department of Comparative Medicine. Female mice aged 6 to 12 weeks were used for these studies. Small sample sizes of 2–6 mice per group were selected for these experiments based on previous experiences and the exploratory nature of the aims. All protocols and experiments were reviewed and approved by the Saint Louis University Animal Care Committee in adherence to AAALAC guidelines and recommendations.

*Leishmania major* parasites were passaged through WT BALB/c mice and maintained in culture in M199 medium for less than 10 passages before use. Infectious metacyclics were isolated using a Ficoll gradient (Spath and Beverley, 2001). Log-phase FV1 parasites were resuspended in DMEM, then added over 10% Ficoll overlaying 20% Ficoll in a sterile tube. After a 15 min spin at 2,500 rpm, the top fraction containing metacyclics was aspirated, washed and resuspended in PBS. For vaccinations, *L. major* parasites in 10 μl PBS were injected intradermally into the hind footpad of mice anesthetized with Ketamine/Xylazine (60 and 5 mg/kg, respectively). The Tulahuèn strain of *T. cruzi* was passaged through *Dipetalogaster maximus* insects and BALB/c mice and maintained in culture in LDNT+ medium (to simulate the foregut) and enriched Grace’s insect medium (to simulate the hindgut). For challenges, *T. cruzi* blood-form trypomastigotes (BFTs) were isolated from the blood of highly infected animals, resuspended in 100 μl of PBS and injected subcutaneously at the base of the tail. Inoculum doses were chosen based on relative virulence of the host strain, titration experiments comparing immunity and pathology resulting after inoculation with various doses (not shown), and prior experience in our lab.

### ELISPOT assays

Total spleen cells (SCs) and popliteal lymph node cells (pLNCs) were isolated from mice *via* mechanical disruption and NH_4_Cl lysis of red blood cells. Mixed cellulose ester bottom filter ELISPOT plates (Millipore) were coated with 10 μg/ml of α-IFN-γ clone R46A2 (BD Pharmingen) and blocked with 10% FBS. Total SCs (3 × 10^5^) were seeded to each well along with 1 × 10^5^ control A20 antigen presenting cells (A20 NC), A20 cells transfected with the TS enzymatic domain (A20 TS), or *L. major* lysate (1 × 10^6^ parasite equivalents/well). After overnight incubation, cells were lysed with H_2_O, and plates were incubated with biotin α-IFN-γ clone XMG1.2 (BD Pharmingen) for 2 h at room temperature, then streptavidin conjugated to horseradish peroxidase for 1 h at room temperature. Spots were visualized through the precipitation of 3-amino-9-ethylcarbazole substrate.

### Antibody ELISA

Serum samples from vaccinated mice were diluted 1:10 in PBS and then serially diluted 1:3. Diluted serum samples were incubated overnight on NUNC Maxisorp plates previously coated in 5 μg/ml of recombinant TS protein and blocked with 10% FBS. The next day, plates were washed and incubated with 1/5000 dilution of rat α-mouse IgG HRP for 2 h at room temperature, then washed and developed with TMB substrate. Reactions were stopped with H_2_SO_4_, and plates were read at 450 nm.

### Intracellular cytokine staining

Cells were cultured with monensin (GolgiPlug, 1 μg/ml) and Brefeldin A (GolgiStop, 0.67 μg/ml) at 37°C for at least 3 h to trap intracellular cytokines. Cells were then fixed and permeabilized with Foxp3/Transcription Factor Staining Buffer Set (eBioscience), then incubated with fluorochrome-conjugated antibodies directed against intracellular cytokines for 30 min at 4°C. After staining, cells were washed and stored in the dark at 4°C until they were acquired by a BD LSR II flow cytometry.

## Results

### Generation of *L. major* parasites expressing a *T. cruzi* TS catalytic domain including derivatives lacking catalytic activity or signal sequences

We selected an active *T. cruzi* TS gene (Costa et al., 1998) and inserted its 678 aa catalytic domain into the strong *Leishmania* expression vector pIR1SAT (Capul et al., 2007a), inserting a CCACC sequence conferring high translatability upstream of the start codon, as described in the methods (Figure 1A). To explore TS secretion when expressed heterologously and its impact on the immune response, we expressed a truncated TS lacking the presumptive 33 amino acid secretory domain, and to explore the requirement for TS enzymatic activity, we engineered a catalytic site inactivating point mutation (Y374H for WT) into both the WT and truncated non-secretory TS. These constructs are termed pIR1SAT-aTS+S (WT TS – active+signal sequence), pIR1SAT-aTS−S (active, lacking signal sequence), pIR1SAT-iTS+S (inactive+signal sequence) or pIR1SAT-iTS−S (inactive lacking signal sequence).

Each construct was digested with SwaI and transfected into *L. major*; using the fully virulent strain FV1, a partially attenuated strain CC1, or the avirulent dihydrofolate reductase, thymidylate synthetase double knock-out CC1 (*dhfr-ts*^−^ /CC1; Figure 1D). SwaI digestion exposes sequences targeting the construct into the parasite rRNA gene locus, where high levels of expression of active mRNA arises from the strong rRNA promoter and processing to mature TS-expressing mRNAs by the parasites’ *trans*-splicing and polyadenylation machinery (Capul et al., 2007a, 2007b). Successful transfection and integration into the rRNA locus were confirmed by PCR tests (not shown) and numerous clonal lines were obtained, of which several were taken for subsequent analysis. Formally these transfectants were termed SSU:IR1SAT-aTS+S, etc., to mark the integration status.

### TcTS expression, activity, and secretion in transfected FV1 *L. major*

**Figure 2:**
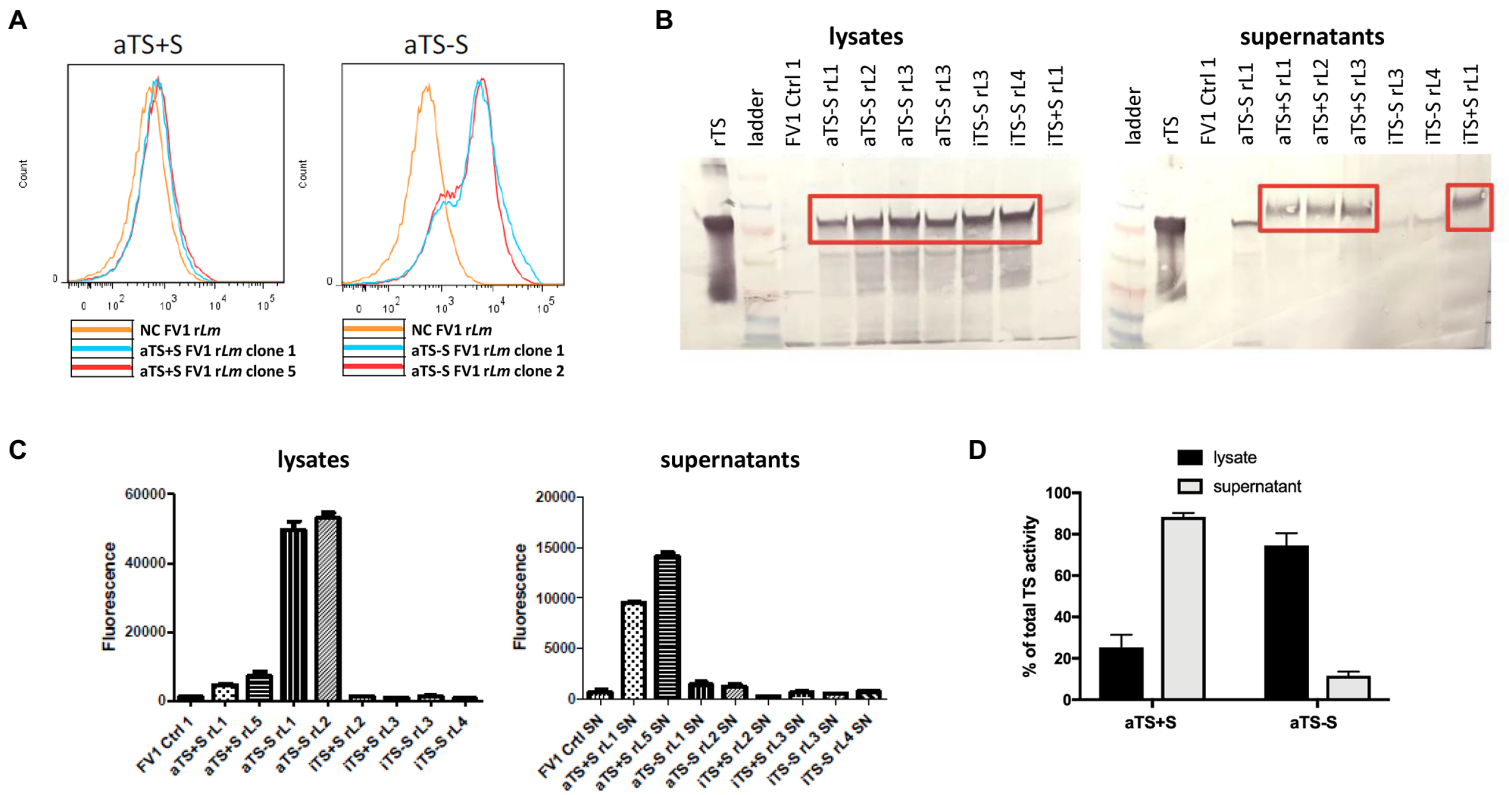
Screening of TS-expressing recombinant FV1 *Leishmania* major parasites. **(A)** After transfection, intracellular TS could be detected by flow cytometry in parasites expressing nonsecreted TS (aTS−S), but not in parasites expressing the secreted form (aTS+S), confirming appropriate deletion or maintenance of the signal sequence. **(B)** As expected, TS protein was detected by Western Blot analysis in the lysates of parasites expressing nonsecreted protein (aTS−S and iTS−S), and in the supernatants of parasites expressing secreted protein (aTS+S and iTS+S). **(C)** Significantly higher levels of enzymatic activity were detected in the lysates of parasites expressing active non-secreted TS (aTS−S) compared to the lysates of parasites expressing inactive non-secreted TS (iTS−S). TS enzymatic activity was similarly higher in the supernatants of parasites expressing active secreted TS (aTS+S) compared to supernatants of those expressing inactive secreted TS (iTS+S). **(D)** Some residual TS activity could be detected in the lysates of aTS+S parasites, and in the supernatants of aTS−S parasites.

We screened the transfected FV1 parasites for the expression of TS using flow cytometry, western blotting, and enzyme activity assays. High levels of intracellular TS expression were detected by flow cytometry and by western blotting within parasites expressing the non-secreted forms of TS (aTS−S; Figures 2A,B). Conversely, much less intracellular TS was detected in parasites expressing Tc TS retaining its native secretion signal (aTS+S; Figures 2A,B). These data confirmed the functionality of the TS signal sequence when expressed in *Leishmania*, and the loss of secretion in its absence.

TS enzymatic activity was determined in parasite cellular lysates or culture supernatants. As expected, enzymatic activity was seen only from transfectants expressing the “active” TS mutants (SSU:IR1SAT-aTS+S, -aTS−S) but not the inactive mutants (Figures 2C,D). For the active TS, activity was seen mostly in the supernatant for the WT secreted TS (SSU:IR1SAT-aTS+S), and mostly in the cell lysate for the WT non-secreted TS (SSU:IR1SAT-aTS−S; Figures 2C,D). These data indicate that our recombinant *Lm* Tc expression constructs predominantly express TcTS with the predicted properties (secreted or not, catalytically active or not).

### Vaccination with live replicating non-attenuated rTS-*Lm* can induce TS-specific T cell responses *in vivo* and protect against virulent challenge

The rTS-*Lm* vaccines based on the parent Friedlin strain FV1 generated above were expected to cause some pathology in mice. However, they also presented us the opportunity to quickly assess the immunogenicity of a persistent leishmania-based *T. cruzi* vaccine in an animal model, even though such a persistent parasite backbone would not be amenable for use in humans. We asked whether expression of the Tc TS forms could induce TS-specific immune responses *in vivo*, and ultimately, protection against a *T. cruzi* challenge. We first vaccinated wild-type BALB/c mice with 1,000 FV1 strain *L. major* parasites transfected with the empty pIR1SAT vector as a negative control (NC), aTS+S, or aTS−S *via* intradermal injection into the hind footpad. Some animals developed severe footpad swelling and required euthanasia (not shown). Representative surviving mice were sacrificed 3 months after vaccination for immune studies. From these mice, total SCs and draining pLNCs were isolated and re-stimulated in IFN-γ ELISPOT assays to measure the development of antigen-specific responses.

**Figure 3:**
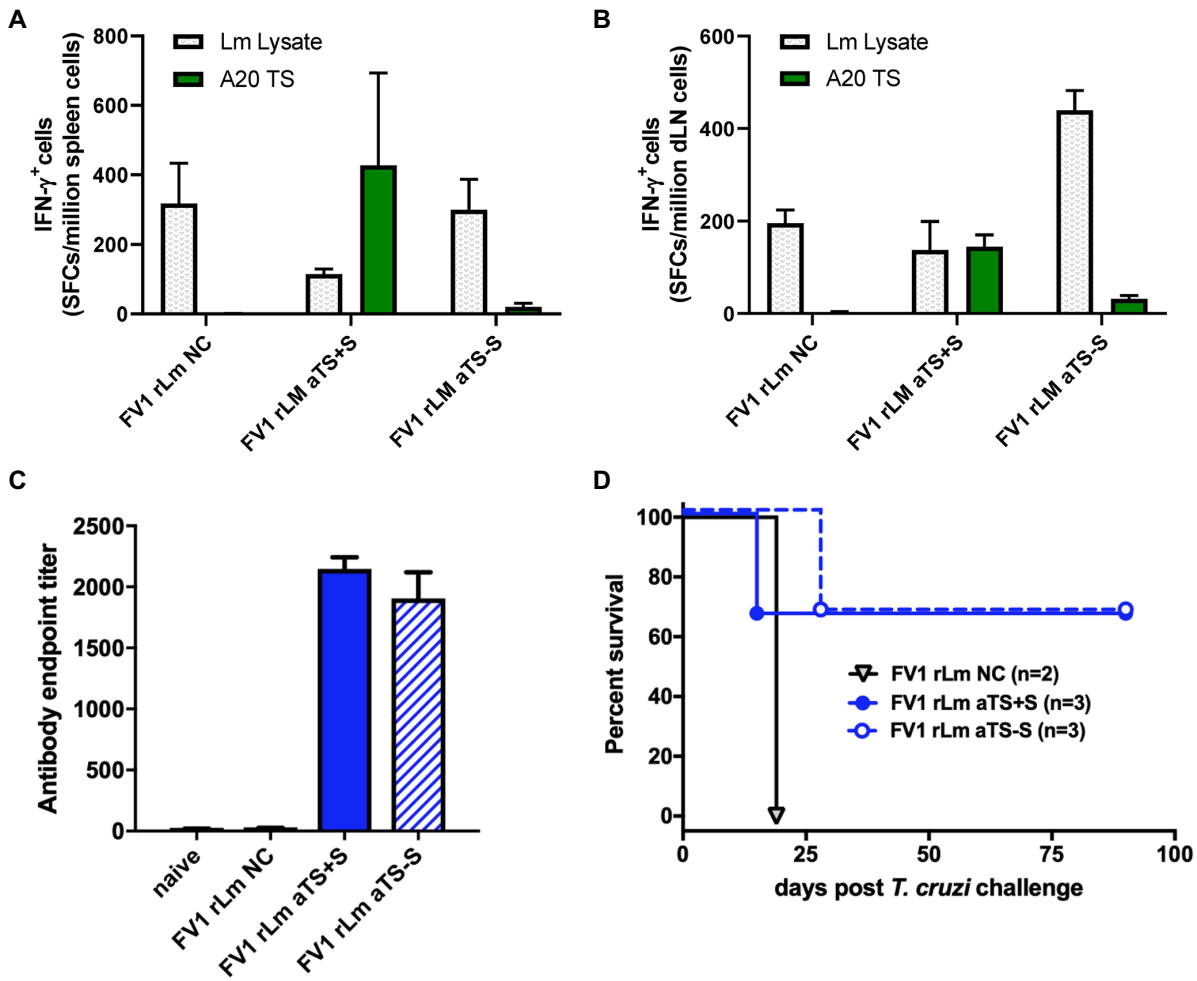
Recombinant TS-expressing FV1 *L. major* parasites induce T cell and antibody responses protective against *T. cruzi* challenge. Mice were inoculated by footpad injection with 1,000 FV1 *L. major* parasites transfected with a control vector (NC), active secreted TS (aTS+S), or active nonsecreted TS (aTS−S). TS-specific IFN-γ responses were detected by ELISPOT assay among spleen cells **(A)** and lymph node cells **(B)** from mice vaccinated with FV1 r*Lm* aTS+S compared to NC, and to a lesser extent from mice vaccinated with FV1 r*Lm* aTS−S. Mice inoculated with FV1 parasites expressing either secreted or non-secreted forms of active TS protein had significantly higher TS-specific antibody titers measured by ELISA compared to control **(C)**, and some mice survived a normally fatal challenge **(D)**. NC A20 - negative control antigen presenting cells; Lm Lysate– *L. major* parasite lysate; TS A20 – A20 cells transfected with TS.

SCs and pLNCs from mice vaccinated with NC r*Lm*, aTS+S r*Lm*, or aTS−S r*Lm* produced IFN-γ in response to co-culture with *L. major* lysate, indicating that r*Lm* vaccination induced *Leishmania-*specific memory T cell responses (Figures 3A,B). In addition, SCs and pLNCs from mice inoculated with aTS+S r*Lm* produced IFN-γ after stimulation with A20 APCs transfected with the TS enzymatic domain of TS, indicating successful induction of TS-specific T cell immunity in these animals (Figures 3A,B). However, TS-specific IFN-γ responses were not produced by SCs from mice vaccinated with either NC r*Lm* or aTS−S r*Lm*, and only minimal IFN-γ responses to TS were detected among the pLNCs of mice given aTS−S r*Lm* (Figures 3A,B).

While only mice inoculated with aTS+S r*Lm* developed large numbers of TS-specific IFN-γ-producing SCs, vaccination with either aTS+S or aTS−S *rLm* resulted in the generation of high titers of TS-specific IgG antibody compared to control vaccination (Figure 3C). These studies demonstrate that vaccination with aTS+S r*Lm* expressing TS can induce T cell and antibody responses to both *Leishmania* antigens and *T. cruzi* TS *in vivo*. Vaccination with aTS−S r*Lm* resulted in similar immune responses to *L. major* and high TS-specific IgG titers, but did not result in substantial numbers of SCs that could produce IFN-γ in response to TS, perhaps because of decreased delivery of TS antigen to draining lymph nodes or induction of alternative inflammatory profiles not measured, such as Th17 cell responses.

To study protection, the remaining r*Lm* vaccinated mice were challenged with 5,000 highly virulent *T. cruzi* blood-form trypomastigotes recovered from infected animals. Two of the three mice vaccinated with either aTS+S r*Lm* or aTS−S r*Lm* survived long-term after *T. cruzi* challenge, compared to none of the mice vaccinated with NC r*Lm*. These results indicate that both TS-expressing vaccines can protect against *T. cruzi* challenge, and suggest a protective role for TS-specific antibodies as the common denominator induced by r*Lm* vaccines (Figures 3C,D). T cell IFN-γ production is an important endpoint, because activated CD8+ T cells are generally considered the ultimate effector cells for the control of *T. cruzi* infection. However, survival among mice vaccinated with aTS−S r*Lm* despite the lack of IFN-γ responses detectable by ELISPOT suggests that alternative mechanisms, such as induction of sufficiently strong TS-specific antibody titers capable of neutralizing blood form trypomastigotes, may be enough to protect against future challenge.

### Attenuated r*Lm* strains can serve as vaccine vectors for *T. cruzi*

Our vaccination experiments using the virulent FV1 r*Lm* demonstrate that recombinant TS-expressing *Lm* parasites can indeed induce protection against *T. cruzi* infection, but this strain retains pathogenicity and causes significant skin lesions at the site of injection, which necessitated the sacrifice of many test mice prior to *T. cruzi* challenge.

The promising experiments above were performed using a virulent r*Lm* background, necessitating the use of a low dose immunization protocol which nonetheless occasionally yielded strong pathology from *L. major* itself. Previous studies have shown that the CC1 strain of *Lm* is less virulent and less likely to cause severe pathology in the host than the FV1 strain (Titus et al., 1995). In addition, a mutant CC1 strain containing a double knock-out for both the dihydrofolate reductase (dhfr) and thymidylate synthetase (ts) genes (*dhfr-ts^−^* CC1) has already been developed and characterized as a safe and effective vaccine against virulent *L. major* infection in mice (Titus et al., 1995).

**Figure 4:**
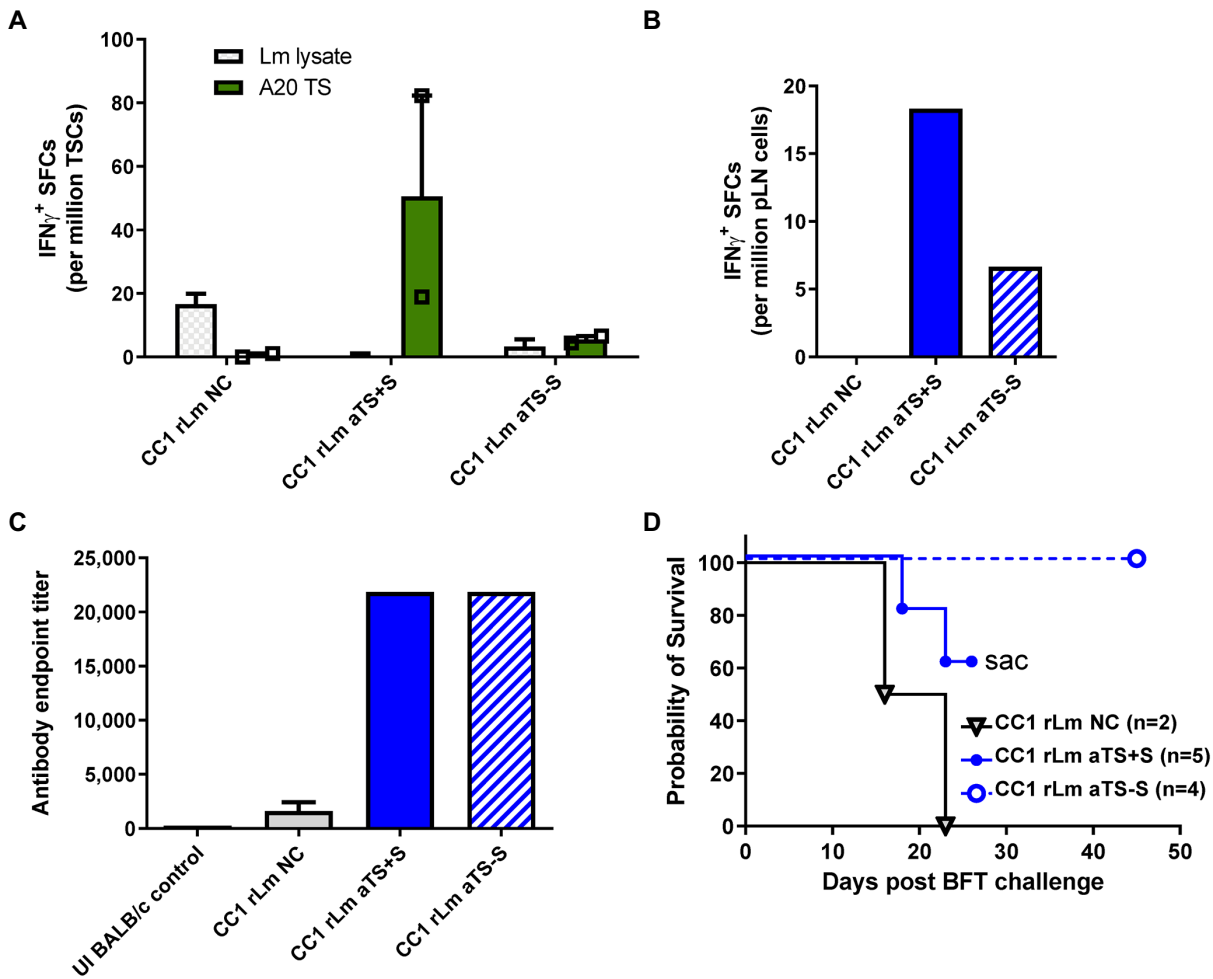
Recombinant TS-expressing CC1 *L. major* parasites induce T cell and antibody responses protective against *T. cruzi* challenge. Mice were inoculated with 10^6^ CC1 *L. major* parasites transfected with a control vector (NC), active secreted TS (aTS+S), or active non-secreted TS (aTS−S). Stronger TS-specific IFN-γ responses were detected on ELISPOT assay among spleen cells **(A)** and lymph node cells **(B)** recovered from mice vaccinated with CC1 r*Lm* aTS+S compared to NC, and to a lesser extent from mice vaccinated with CC1 r*Lm* aTS−S. Mice inoculated with CC1 parasites expressing either form of active TS protein had significantly higher TS-specific antibody titers compared to control, when measured by ELISA assay **(C)**. Mice vaccinated with CC1 rLm aTS−S survived after a normally fatal challenge **(D)**. Mice vaccinated with CC1 rLm aTS+S also survived longer than control mice, but were euthanized due to the development of severe necrotizing lesions at the inoculation site **(D)**.

To test whether the CC1 background on which the *dfr-ts-* mutant exists can also serve as an effective vehicle for TS vaccination, we inoculated mice in the hind footpad with 10^6^ CC1 NC, aTS+S, or aTS−S r*Lm* parasites. This dose was chosen as it led to little Leishmania-induced pathology (data not shown). Approximately one month afterward, representative mice from each group were sacrificed for immune studies. TS-specific IFN-γ T cell responses were detected in both SCs (Figure 4A) and pLNCs (Figure 4B) of mice vaccinated with either aTS+S or aTS−S r*Lm* compared to those vaccinated with NC r*Lm,* although stronger T cell responses were detected in mice vaccinated with aTS+S r*Lm*, consistent with the FV1 vaccination data. However, both aTS+S r*Lm* and aTS−S r*Lm* generated similarly high TS-specific antibody titers (Figure 4C), again consistent with the FV1 vaccination data. Surprisingly, despite inducing lower TS-specific IFN-γ responses compared with aTS+S r*Lm* vaccination, aTS−S r*Lm* conferred the greatest survival benefit following a normally lethal *T. cruzi* challenge (Figure 4D), suggesting that other responses not measured, including Th17 type cytokines, may mediate protection. In contrast, a trend for increased immunopathology in aTS+S r*Lm-*vaccinated animals was seen, as some mice needed to be humanely euthanized due to severe necrotizing lesions at the r*Lm* inoculation site.

### Exploration of more attenuated r*Lm* strains as potential vaccine vectors for *T. cruzi*

Because of the increased safety profile of the double knock-out, mutant *dhfr-ts^−^ Lm* strain, it has the most potential to be applicable in humans. To test the *dhfr-ts^−^* r*Lm* as *T. cruzi* vaccines, we inoculated WT BALB/c mice with 10^6^ log-phase WT or *dhfr-ts^−^* CC1 NC or aTS+S parasites by injection into the hind footpad. Approximately 5 weeks later, representative mice were euthanized for immune studies. No IFN-γ responses to TS stimulation were detected by ELISPOT assay among immune cell populations isolated from mice vaccinated with *dfhr-ts-* r*Lm* expressing NC or aTS+S (not shown).

**Figure 5:**
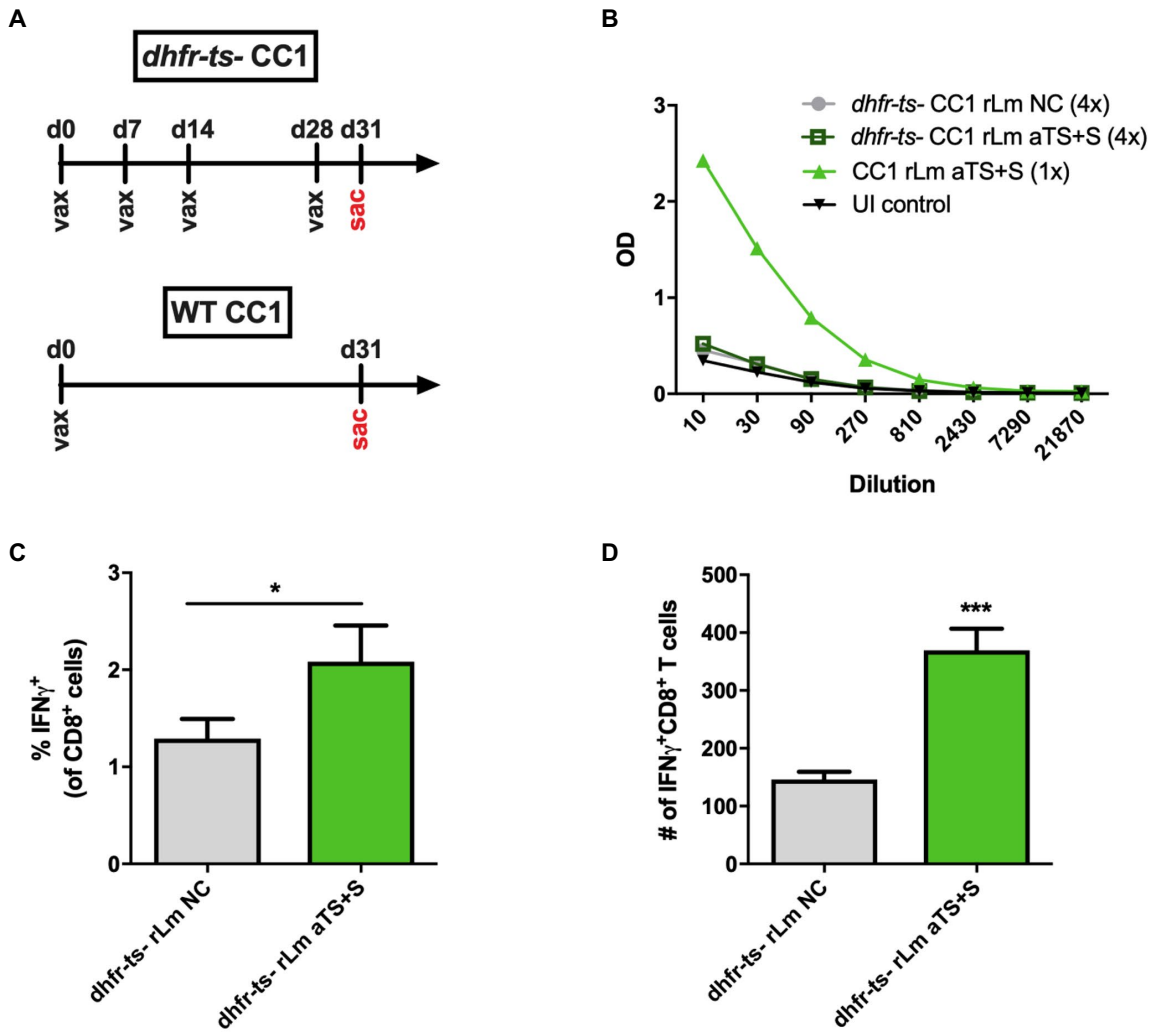
Recombinant TS-expressing *dhfr-ts^−^ -* CC1 *L. major* parasites are less immunogenic compared to virulent recombinants. **(A)** Mice were inoculated four times with 10^6^ attenuated *dhfr-ts^−^* CC1 *L. major* parasites transfected with a control vector (NC) or secreted TS (aTS+S). Some mice were inoculated once with 10^6^ WT CC1 r*Lm* aTS+S as a positive control. **(B)** Mice inoculated with *dhfr-ts^−^* r*Lm* expressing aTS+S did not have significantly higher antibody titers compared to mice inoculated with control *dhfr-ts^−^* r*Lm* parasites. However, on ICS assay, greater percentages **(C)** and numbers **(D)** of spleen cells recovered from mice vaccinated with *dhfr-ts^−^* r*Lm* aTS+S expressed IFN-γ in response to re-stimulation, compared to mice vaccinated with control parasites.

In our experiments, *dhfr-ts^−^* CC1 r*Lm* parasites could be recovered from the footpads of vaccinated animals on day 3 after injection but not on day 7, when WT CC1 parasites are routinely recovered, indicating a limited timeframe of *dhfr-ts^−^* CC1 r*Lm* parasite persistence as described previously (Titus et al., 1995). We hypothesized that the limited persistence of *dhfr-ts^−^* CC1 r*Lm* parasites may limit the amount of TS antigen secreted and able to prime relevant immune cells, so we investigated a prime-boost vaccination strategy to amplify TS-specific immune responses. We vaccinated mice with 10^6^ log-phase *dhfr-ts^−^* CC1 NC or *dhfr-ts^−^* aTS+S CC1 parasites once a week for 3 weeks, then boosted these mice with another dose 3 days prior to sacrifice at 4 weeks for immune studies (Figure 5A). Repeated prime-boost vaccination with *dhfr-ts^−^* aTS + S did not generate high titers of TS-specific IgG antibodies compared to vaccination with control parasites (Figure 5B). We also did not detect any TS-specific responses among SCs by IFN-γ ELISPOT assays. However, in ICS assays, we measured some increased expression of IFN-γ among CD8+ T cells recovered from mice vaccinated with *dhfr-ts^−^* aTS+S r*Lm* (Figures 5C,D).

None of the mice vaccinated with *dhfr-ts^−^* CC1 r*Lm* developed lesions despite multiple injections, consistent with prior studies where very high inocula were required (Titus et al., 1995), while all mice receiving WT CC1 vaccination developed significant swelling within 2 months, with some developing ulcerative lesions (although not to the degree seen with FV1 infections). Thus, we confirmed the avirulent and nonpathogenic nature of the *dhfr-ts^−^* CC1 strain compared to the WT CC1 strain. However, the data suggest this strain is not immunogenic enough to induce responses to the recombinant *T. cruzi* antigen that it encodes. The results of these experiments indicate that future work should explore methods of increasing the immunogenicity of the partially attenuated *dhfr-ts^−^* CC1 strain to render it both a safe and effective vehicle for the delivery of vaccine antigens.

### Expression of active TcTS enhances the virulence of *L. major*

**Figure 6:**
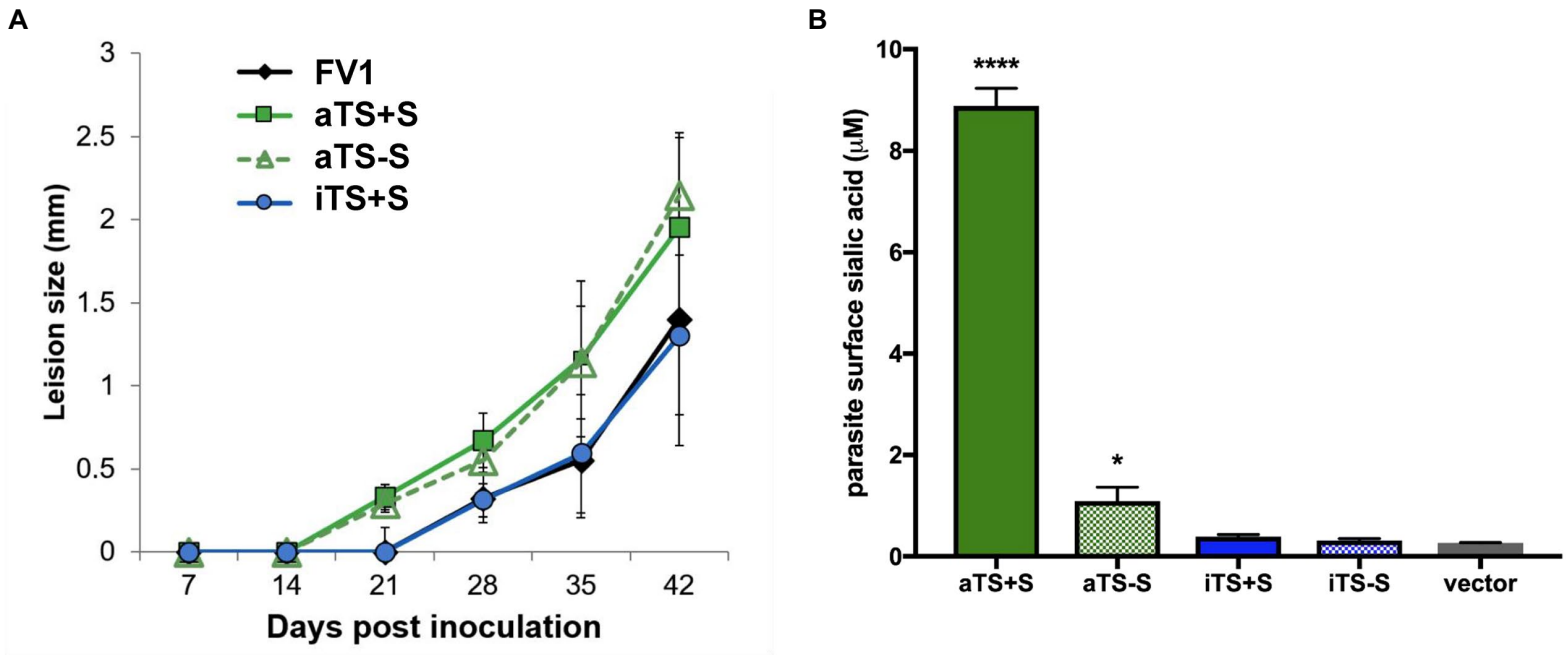
Expression of active TS enhances *Leishmania* virulence. Mice were inoculated by footpad injection with FV1 *L. major* parasites transfected with a control vector (NC), active secreted TS (aTS+S), active nonsecreted TS (aTS−S), or inactive secreted TS (iTS+S). **(A)** Footpad caliper measurements demonstrated that *L. major* parasites expressing active forms of TS caused footpad lesions approximately 7 days earlier than *L. major* parasites expressing inactive TS or control vector. **(B)** Earlier lesion development in mice inoculated with parasites expressing active TS was associated with surface sialylation of these parasites.

We inoculated mice with equal numbers of WT control *L. major* FV1 parasites or recombinant parasites expressing the aTS+S, aTS−S, or iTS+S enzymes. Mice inoculated with r*Lm* expressing active TS, regardless of whether the TS was secreted or non-secreted, developed lesions 1 week earlier than mice injected with WT *L. major* not expressing TS (day 14 compared to day 21, Figure 6A). After lesions appeared, however, the progression was not significantly different between the groups. The kinetics of lesion development were associated with increased expression of sialic acid on the surface of promastigote parasites expressing active TS (Figure 6B). Although the surface sialylation was studied on promastigote recombinants, we expect similar sialylation on amastigotes because of the nature of the expression system used. In contrast, mice inoculated with iTS+S r*Lm* only developed lesions on day 21, similar to WT FV1 parasites, indicating that the expression of enzymatically inactive versions of TS does not result in earlier lesion development.

## Discussion

The development of a human Chagas disease vaccine could yield tremendous benefits for global public health and is a goal that has been pursued for decades. The causative *T. cruzi* parasite expresses several proteins that are promising potential vaccine antigens, including *trans*-sialidase, a protein with a domain that is highly conserved among *T. cruzi* strains and contains protective CD4+ and CD8+ T cell epitopes (Cai et al., 2016). Concomitant infection with *T. cruzi* generates robust immunity against subsequent challenges (Mann et al., 2020), suggesting that live vaccination with attenuated parasites may be optimal for Chagas disease vaccine development. We did not determine whether r*Lm*+TS vaccinated mice surviving *T. cruzi* challenge harbored low level tissue or blood amastigotes, thus future studies should evaluate this more fully. In animal models, reduction of parasite burden by drug therapy can prevent progression to more severe pathology, indicating that even a non-sterilizing vaccine could provide significant clinical benefits (Garcia et al., 2005). *T. cruzi*-specific CD8^+^ T cell responses have roles in both protection and immunopathology. We identified TS-specific CD8^+^ T cell responses induced by r*Lm*+TS infection, however, the exact role of these T cells in our model still needs to be determined.

A naturally attenuated strain of *T. cruzi* has been tested as a Chagas disease vaccine in animal models (Basombrio et al., 1993; Perez Brandan et al., 2011), but the possibility of reversion to virulence *in vivo* raises important safety concerns limiting the development of such strains for human vaccination. Pathogens that have been genetically attenuated by the ablation of two independent genes are less likely to revert to virulence than naturally attenuated strains. This genetic attenuation has been successfully performed with the related trypanosomatid *Leishmania major via* deletion of the bifunctional dihydrofolate reductase-thymidylate synthase gene (*DHFR-TS*), whose enzymatic producted is required for thymidine and amino acid synthesis (Titus et al., 1995). These mutations result in an auxotrophic mutant (*dhfr-ts*^−^) that cannot replicate nor survive long-term, and does not cause disease *in vivo* (Gueiros-Filho and Beverley, 1994). Previous research has demonstrated that inoculating mice with the *dhfr-ts^−^* CC1 strain of *L. major* confers protective immunity against future infection with virulent *L. major* strains, confirming its promise as an experimental vaccine for leishmaniasis. Similar attempts to generate *dhfr-ts^−^ T. cruzi* have been challenged by the inability to generate homozygous deletions (Perez Brandan et al., 2011). Thus, we asked whether we could use the pre-existing *dhfr-ts^−^* CC1 *L. major* as a live vehicle for the delivery of Chagas vaccine antigens by genetically engineering the parasites to express an immunogenic *T. cruzi* protein.

For these studies, we cloned various forms of the *T. cruzi* TS domain into the virulent FV1 strain, the less virulent CC1 strain, and the avirulent *dhfr-ts^−^* CC1 strain of *L. major* and screened the recombinants by flow cytometry, western blot and enzyme activity assay. *In vivo*, we found that inoculation of mice with FV1 rTS-*Lm* induced TS-specific T cell and antibody responses and conferred a survival advantage after a subsequent challenge with a normally lethal dose of virulent *T. cruzi.* Increased TS-specific T cell and antibody responses could be similarly detected after vaccination of mice with the CC1 strain. However, TS-specific protective immune responses were not induced by the avirulent, attenuated *dhfr-ts^−^* CC1 r*Lm*.

Previous studies reported that the expression of a form of TS increased the virulence of a partially virulent line of *L. major* parasites (Belen Carrillo et al., 2000), raising some safety concerns. However, in that study, the parasites expressed a full-length active TcTS, including both the catalytic domain and highly immunogenic repeated sequence region. This left open the question of whether TS activity and/or immune response were responsible. Here we expressed only the TS catalytic domain, and the data confirmed that increased virulence could associate with active TS expression. In contrast, parasites expressing an inactive form of TS developed lesions later, suggesting less potential for pathogenicity. If recombinant inactive TS is shown to be as immunogenic as enzymatically active versions of the enzyme, the expression of inactive TS may be a safer approach to consider when developing live parasite vaccine vehicles in the future. Fontanella et al. (2008) also confirmed the immunogenicity and protection using purified catalytically inactive protein.

The major advance with this work is the evidence that *Leishmania major* parasites can serve as a live vaccine vector. Bacterial species including *Salmonella, Listeria, Yersinia* and *Mycobacterium* are more commonly used for the delivery of foreign vaccine antigens by live vectors (da Silva et al., 2014). We previously demonstrated that *Salmonella* expressing the *T. cruzi* cruzipain protein can provide some protection against *T. cruzi* infection (Schnapp et al., 2002). Other groups have demonstrated protection using recombinant Salmonella vaccines encoding the Tc52 antigen (Matos et al., 2014) and *Mycobacterium bovis* BCG encoding TS (Bontempi et al., 2020). However, the use of a related protozoan species such as *Leishmania* can potentially better mimic natural *T. cruzi* infection. *Leishmania* parasites also exhibit strong tropism for phagocytic cells, such as macrophages and dendritic cells, which serve as effective antigen presenting cells. Therefore, *Leishmania* as a live vector could facilitate the efficient delivery of vaccine antigens to relevant cells. Finally, rTS-*Lm* vaccines have the potential to induce protective immunity against both *T. cruzi* and *L. major*, two significant human pathogens that can present as co-infections in endemic areas.

In summary, we demonstrate that *L. major* parasites expressing a recombinant *T. cruzi* antigen can confer protection against future infection with virulent *T. cruzi.* These preliminary studies warrant further investigation into the use of *Leishmania* as an antigen delivery system. Because the *dhfr-ts^−^* mutant has little chance of reverting to virulence, this strain presents the greatest potential for translation into clinical applications. However, we did not observe evidence of protective immunity generated using *dhfr-ts-* recombinants, including with a prime-boost approach, which is a limitation of the current study. Therefore, future work should focus on increasing the immunogenicity of this attenuated strain, such as by optimizing the prime-boost schedule or by biasing for specific immune responses, possibly with the addition of adjuvants biasing for Th1 or Th17 cell phenotypes, shown to be protective against *T. cruzi* (Cai et al., 2016) or the genetic manipulation of *L. major* vectors to co-express IL-12 or other cytokines. Finally, any future work should focus on exploring recombinants expressing inactive TS, which may be safer than recombinants expressing active forms of the virulence factor. With increasing understanding and characterization, the usage of recombinant *dhfr-ts^−^ L. major* parasites to deliver foreign antigens could prove to be an effective strategy for vaccine development for *T. cruzi* and other infectious pathogens.

## Data availability statement

The original contributions presented in the study are included in the article further inquiries can be directed to the corresponding author/s.

## Ethics statement

Animal studies were reviewed and approved by the Saint Louis University Institutional Animal Care and Use Committee.

## Author contributions

DH, SB, and CC contributed to the conception and design of the study. CC, AO’S, CE, HG, and WL designed and performed experiments and analyzed the data. CC prepared the first draft of the manuscript. All authors contributed to manuscript revisions.

## Funding

This work was supported by NIH grants AI128270 (DH), AI031078 (HG, SB), and AI29646 (SB).

## Conflict of interest

The authors declare that the research was conducted in the absence of any commercial or financial relationships that could be construed as a potential conflict of interest.

## Publisher’s note

All claims expressed in this article are solely those of the authors and do not necessarily represent those of their affiliated organizations, or those of the publisher, the editors and the reviewers. Any product that may be evaluated in this article, or claim that may be made by its manufacturer, is not guaranteed or endorsed by the publisher.
